# Current pesticide dietary risk assessment in light of comparable animal study NOAELs after chronic and short-termed exposure durations

**DOI:** 10.1007/s00204-017-2052-4

**Published:** 2017-09-19

**Authors:** Jürg A. Zarn, Caitlyn D. O’Brien

**Affiliations:** Federal Food Safety and Veterinary Office (FSVO), Risk Assessment Division, Schwarzenburgstrasse 155, 3003 Bern, Switzerland

**Keywords:** Dietary risk assessment, Intermittent exposure, NOAEL, Pesticides, Time extrapolation

## Abstract

Dietary risk assessment (DRA) of pesticides includes the estimation of chronic and acute exposures from crop residues, but assesses acute exposures only for pesticides with an acute reference dose (ARfD). Acute estimation uses high percentiles of food consumption surveys which are considerably higher than per capita lifetime averaged food consumption values which are used for chronic estimations. Assessing acute risks only for pesticides with an ARfD tacitly assumes that chronic risk assessment covers also intermittent occurring exposures which could significantly exceed chronic estimates. The present investigation conducted on 2200 rat studies from 436 pesticides provides evidence demonstrating that pesticides with and without ARfD have no-observed-adverse-effect levels (NOAELs) which remain statistically unchanged in developmental, subacute, subchronic, reproductive and chronic toxicity studies covering exposure durations between 2 and 104 weeks. DRA of pesticides without ARfD needs reconsideration in light of equally high toxic dose levels after short- and long-term exposures, suggesting that intermittent exposures could be toxic, if they repeatedly exceed the acceptable chronic daily intake (ADI; conceptually the human counterpart of chronic animal NOAEL). As such risks are currently not assessed for pesticides without ARfD, the current DRA concept, which automatically presumes the use of low chronic exposure estimates entirely covers the risks of not acutely toxic pesticides, needs reconsideration. Furthermore, risks to intermittent occurring high exposures are probably also insufficiently assessed for pesticides where the ARfD is significantly higher than the ADI. As an example, the maximum residue limit for bifenazate in peaches is discussed.

**Electronic supplementary material:**

The online version of this article (doi:10.1007/s00204-017-2052-4) contains supplementary material, which is available to authorized users.

## Introduction

Dietary risk assessment (DRA) is an integral part of the authorization process of plant protection products (PPP), within which exposure risks related to residues remaining in treated crops are assessed. DRA compares estimates of acute and chronic exposures of the compound to human health-based guidance values (HBGVs). DRA is conducted for the purpose of defining legally binding safe maximum residue limits (MRLs) for each pesticide–crop pair. For this purpose, DRA needs to adequately integrate the toxic potency and the exposure for different exposure durations. HBGVs such as acute reference dose (ARfD), acceptable daily intake (ADI) and acceptable operator exposure level (AOEL) are derived from toxicological data and represent maximum acceptable exposure limits for humans at different exposure scenarios (FAO/WHO 2009).

The ARfD expresses the maximum dose one may ingest on a single day, whereas the ADI is the maximum daily dose one may ingest one’s whole life, each without an appreciable health risk. The AOEL corresponds to the short-term HBGV for operators and workers exposed only over a short time (EU 2006).

For the purpose of DRA, the HBGVs ARfD and ADI are applied. The current international guidance (OECD 2010) on setting ARfDs refers to Solecki et al. (2005), which states that ARfDs must be equal to or higher than ADIs. According to these guidance documents, an ARfD should be established only if there is evidence that a 1-day exposure might induce adverse effects.

Although the mandatory toxicology data package submitted for the approval of pesticides was not developed to identify adverse single-dose effects, it is nevertheless believed to be sufficiently sensitive to identify such effects. A retrospective analysis showed that ARfDs are typically established on the basis of fetal toxicity, teratogenicity or maternal toxicity observed in developmental toxicity studies or clinical observations in acute neurotoxicity studies in rats (Solecki et al. 2010). Regulatory bodies estimate dietary exposure by basically multiplying the residue concentration of a pesticide in a crop by appropriate (acute or chronic) consumption levels of the respective crop. The concentrations used are either the pesticide residues measured in crops or the MRLs assigned to each crop–pesticide pair. DRA should warrant that approved MRLs are safe, despite different consumption habits of the population. Huge differences in the consumption of certain food commodities may occur due to seasonal availability of the commodity in question or changes in consumption habits during life stages (milk consumption in childhood). For example, the availability of different kinds of berries and stone fruits (example of peaches as given in “An illustrative example: bifenazate MRL in peaches”) depends upon the season, which might lead to a predominant consumption by fanciers during the season in contrast to virtually no consumption during the off-season.

For this purpose, the current system of DRA applies two separate toxicological concepts for the assessment of acute and chronic exposure estimates, noting that the World Health Organization (WHO 2015) and the EU (EFSA 2016) basically follow similar procedures in their calculations. For the estimation of acute exposures, the international estimated short-term intake (IESTI) model is applied, which bases exposures upon high food intake percentiles from food consumption surveys. For the estimation of chronic exposures, the international estimated daily intake (IEDI) model is applied, at which exposures are estimated on the basis of considerably lower averaged per capita food consumption data.

The time frame for acute exposure is 24 h, whereas the time frame for chronic exposure is not clearly defined, but is generally understood to be longer than 90 days. An exposure model safeguarding against exposures shorter than chronic but longer than 1 day currently does not exist. For compounds regarded as not acutely toxic, no ARfD is established. Currently, DRA for such compounds includes only a chronic risk assessment, i.e, a comparison of the ADI with low, chronic exposure levels, whereas scenarios with intermittent higher levels of exposures are not considered. The risk of intermittent higher exposures is considered to be covered by chronic scenarios, obviously because it is assumed, that the toxic potency increases with exposure duration, which would be tantamount to short-term NOAELs being significantly higher than chronic NOAELs.

A wealth of literature exists that has investigated the relationship between NOAELs and study duration (Batke et al. 2011; Bitsch et al. 2006; Bokkers and Slob 2005; Doe et al. 2006; Dourson et al. 1992; Groeneveld et al. 2004; Pieters et al. 1998; Pohl et al. 2009; Woutersen et al. 1984). In general, most of these evaluations have concluded that shorter-term studies have higher NOAELs than longer-term studies. Still certain study design factors were demonstrated to lead to a non-toxicological distortion of NOAEL distributions, such as number of animals per group, dose spacing and dose decrement in feeding studies. When these study design factors are taken into account, NOAELs derived from subacute (4 weeks), subchronic (13 weeks) and chronic studies (1 year or more) are not significantly different (Zarn et al. 2010, 2011). In other words, when the NOAELs are corrected for study design factors, compounds reveal comparable potencies after short-term and chronic exposures. Despite this fact, PPP without an ARfD are only chronically assed on the basis of low chronic food consumption values. Using low exposure estimates presumes that higher exposure levels, which could even exceed chronic exposure levels, are sufficiently covered by the chronic risk assessment as they have a shorter duration. Bearing in mind comparable potencies after short-term and chronic exposures, this assumption at least is disputable. As an example of a possible health concern the current MRL for bifenazate in peaches is given in “An illustrative example: bifenazate MRL in peaches”, which illustrates that health risks might emerge from intermittent higher exposures and are not assessed for not acutely toxic PPP within the current approach of DRA. The aim of the present study was to confirm whether the current DRA approach offers sufficient protection to short-termed exposures which exceed chronic exposure levels when PPP with no ARfD or an ARfD significantly higher than the ADI are assessed. Omitting such a scenario, as it is currently done for a PPP without ARfD, assumes a low probability for such PPP to provoke adverse effects, when the exposure duration is short. Commensurate to this assumption, a lower toxic potency at shorter exposure durations would come along with short-term NOAELs being significantly higher than long-term NOAELs. Our investigations therefore focused on the question, if the toxic potency of PPPs without ARfDs increases with longer exposure durations (i.e, whether PPP without ARfD have short-term NOAELs being significantly higher than long-term NOAELs).

As a measure, ratios of NOAELs (and lowest-observed-adverse-effect levels [LOAELs]) from shorter than chronic studies (<104 weeks) to chronic NOAELs (and LOAELs) (104 weeks) were analyzed. Since an increasing toxic potency with longer exposure durations would mean progressively lower NOAELs with longer exposure durations, the ratio would gradually shift towards 1, when NOAELs (and LOAELs) of increasing study duration are consecutively compared to chronic NOAELs (LOAELs). Throughout the study we also focused on significant differences between PPPs with and without an ARfD regarding the relationship between short-term and chronic toxic potencies.

## Methods

### Database

Toxicity data were managed by means of a Microsoft Office Access 2007 database (Zarn et al. 2011, 2015) populated with publicly available PPP evaluations, which were retrieved from the European Food Safety Authority (EFSA 2014), the Joint FAO/WHO Meeting on Pesticide Residues (JMPR) (WHO 2011) and the United States Environmental Protection Agency (USEPA 2011). Extracted data include the chemical class of the compound as well as study-specific data, such as species and strain, number of animals per group, exposure duration, application route, dose levels, and the NOAEL and LOAEL reported by the evaluating authority (see also file “Supplemental Data”).

The present evaluation considered only rat studies conducted by the oral administration route (i.e., feeding and gavage studies) in which an experimental NOAEL and LOAEL was identified. The ratio calculations reported here are derived only from pairs of NOAELs (or pairs of LOAELs) retrieved from the same authority. In the rare case in which more than one suitable study was available, the study with the lowest dose spacing (i.e., lowest ratio of LOAEL to NOAEL within the study) was used. If more than one evaluation was available for a certain PPP, the selection followed the order EFSA > JMPR > US EPA the same order followed in previous evaluations (Zarn et al. 2011, 2013).

Previously reported analyses of NOAEL ratio distributions (Zarn et al. 2010, 2011) included subacute, subchronic and chronic studies with exposure durations of approximately 4, 13 and 104 weeks, respectively (OECD 1998, 2008, 2009a, b, c). The study described in this paper expands the scope of previous analyses by additionally including developmental and reproductive toxicity (multigeneration) studies (OECD 2001a, b). In developmental toxicity studies in rats, the typical exposure duration is 2 weeks, from gestation days 6–20, with study termination at the time of the removal of the fetuses by cesarean section. Typical reproductive toxicity studies in rats have an average exposure duration of 18 weeks, including 10–14 weeks in the premating phase and about 3 weeks in each of the gestation and lactation phases. As developmental and reproductive toxicity studies rarely include hematological, clinical chemistry or histological endpoints, these studies are less sensitive than subacute, subchronic and chronic studies with respect to these endpoints. In contrast, developmental and reproductive toxicity studies typically involve 20–30 animals per dose group, at least twice as many animals as in subchronic studies. Therefore, developmental and reproductive toxicity studies may be less sensitive with respect to the parameters investigated in parental animals, but still have a statistical power that lies somewhere between those of subchronic and chronic studies.

Overall, the evaluation described in this paper includes NOAELs derived from rat studies with exposure durations of 2, 4, 13, 18 and 104 weeks.

### Study design factors affecting NOAEL ratio distribution

The resulting database is referred to as the complete data set (CS). In order to account for the distorting effect of high dose spacing on the NOAEL ratio distribution, we excluded studies with dose spacing greater than 5 or greater than 8 for certain analyses, terming these subsets CS5 and CS8, respectively.

Furthermore we considered dose decrement in feeding studies, which was shown to have distorting effects on analyses of NOAEL ratio distributions in former analyses (Zarn et al. 2013). Dose decrement refers to the steadily decreasing administered doses in feeding studies, since the animals’ intake of feed per body weight steadily decreases during the course of a study, whereas the concentration in feed is usually not adjusted for this decrease. If procurable, the NOAELs were, therefore, expressed as concentration of the pesticide in the feed (e.g., ppm).

For more details on the database, the selection criteria and the generated subsets, the reader is referred to previous publications that used similar criteria (Zarn et al. 2011, 2015).

### Data analyses

The database included relevant data on 436 pesticides. ARfDs were established for 295 (68%) of these pesticides as part of their evaluation by EFSA, JMPR or the US EPA. Data processing and statistical analyses were conducted by means of Microsoft Office Access 2007, Microsoft Office Excel 2007 and IBM SPSS Statistics 22 for Windows. In a first analysis, we queried the database methodologically as described by Zarn et al. (2015) for the lowest NOAEL available to each compound, hereby distinguishing between PPPs with and without an ARfD (see “Distributions of the lowest NOAELs for PPPs with and without an ARfD”).

Statistical analyses investigate the following ratios of NOAEL or LOAEL values: developmental to chronic, subacute to chronic, subchronic to chronic, and reproductive to chronic (see “Influence of exposure duration on NOAEL and LOAEL ratio distributions”). For developmental and reproductive studies only parental effect levels were used, as parental NOAELs are usually lower than fetal developmental, respectively, offspring reproductive NOAELs. To elucidate differences between compounds with and without ARfD we divided the ratios into groups of compounds with and without an ARfD (see “Distributions of NOAEL and LOAEL ratios for compounds with and without an ARfD”).

Statistical comparisons using the Kruskal–Wallis test with Mann–Whitney *U* as post hoc test were subsequently performed following in two approaches: (1) based on a certain dose spacing cut-off level (e.g., CS8) three different groups of distributions (“all compounds”, “compounds with an ARfD” and “compounds without an ARfD”) of a certain NOAEL ratio (e.g., subacute to chronic) were compared and analyzed for statistically significant differences; (2) Based on a certain dose spacing cut-off level (e.g., CS8) three different distribution groups “all compounds”, “compounds with an ARfD” and “compounds without an ARfD” of different NOAEL ratios—developmental (parental) to chronic, subacute to chronic, subchronic to chronic, and reproductive (parental) to chronic—were compared and analyzed for statistically significant differences. We additionally compared safe acute (ARfD), short-term (AOEL) and chronic (ADI) doses for humans by calculating ratios of ARfD to ADI (for the 295 compounds with an ARfD) and ratios of AOEL to ADI (for the 419 compounds with both an ADI and an AOEL) (see “Relationship among ARfD, AOEL and ADI”). For approximately 9% of the AOELs and 14% of the ARfDs, the safety factors applied to derive the respective HBGV are different compared to the safety factors used to derive the ADIs. However, for the vast majority of these different safety factors, the difference was equal or less than threefold.

The outcomes of these analyses are discussed in the context of current paradigms on which DRA and acute and chronic exposure estimation are based on.

## Results

### Distributions of the lowest NOAELs for PPPs with and without an ARfD

**Fig. 1:**
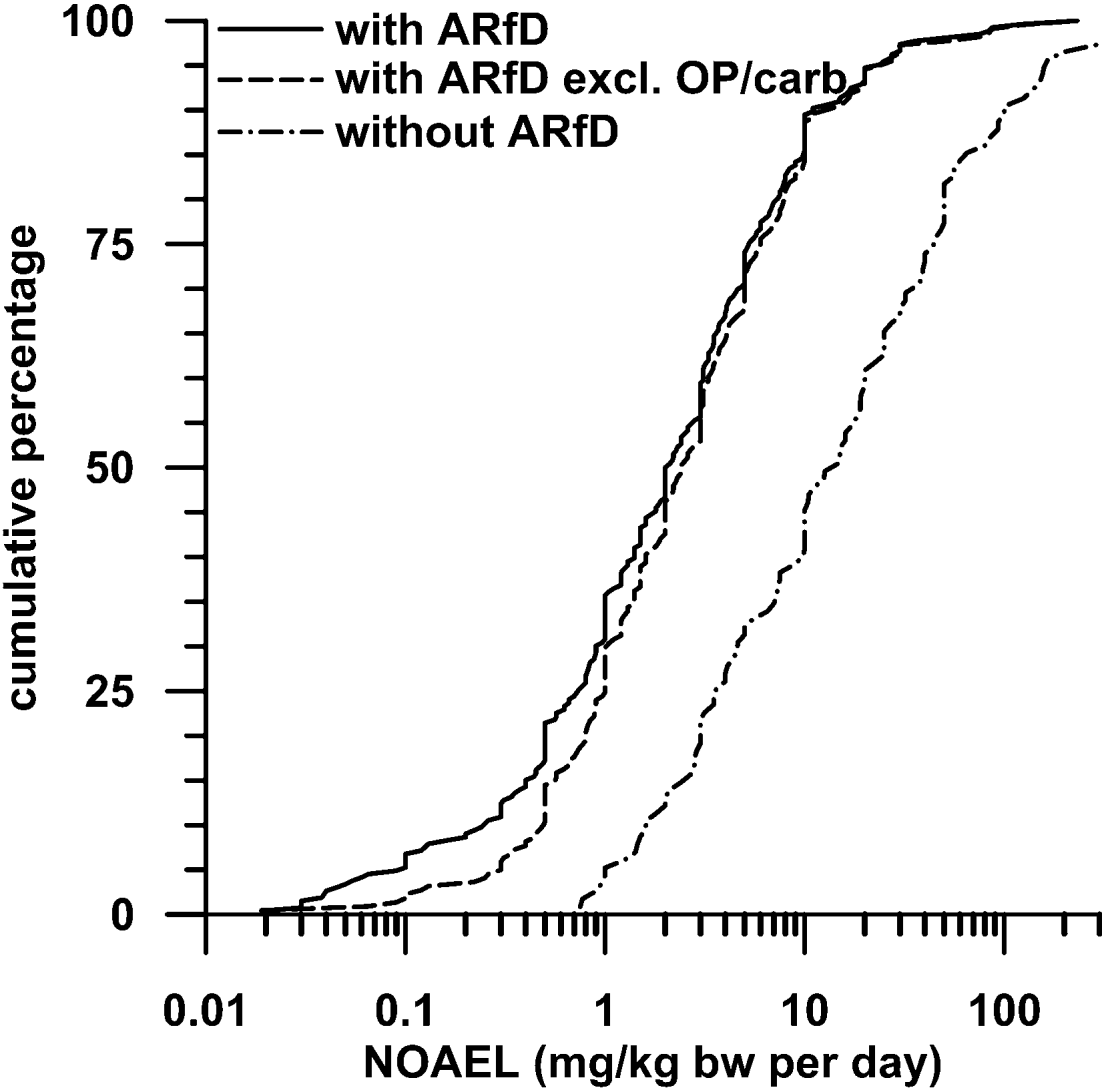
Cumulative distributions of lowest NOAELs for pesticides. The distributions of segregated subsets are presented: pesticides with an ARfD; pesticides with an ARfD but excluding organophosphates (OP) and carbamates (carb); pesticides without an ARfD

Figure 1 shows cumulative distributions of the lowest available NOAELs for each PPP, hereby distinguishing PPP with and without ARfD. As high dose spacing can have an impact on NOAEL and LOAEL distributions (Zarn et al. 2011, 2015), data from studies with a dose spacing greater than 8 (CS8) were excluded.

It is evident that compounds with an ARfD have NOAELs approximately one order of magnitude lower than the NOAELs for compounds without an ARfD. This distinction suggests that compounds with an ARfD are generally more toxic than compounds without an ARfD. When comparing the 5th to 95th percentiles of the distributions for compounds with and without an ARfD, the NOAELs for both groups of compounds cover a range of more than two orders of magnitude.

### Influence of exposure duration on NOAEL and LOAEL ratio distributions

**Fig. 2:**
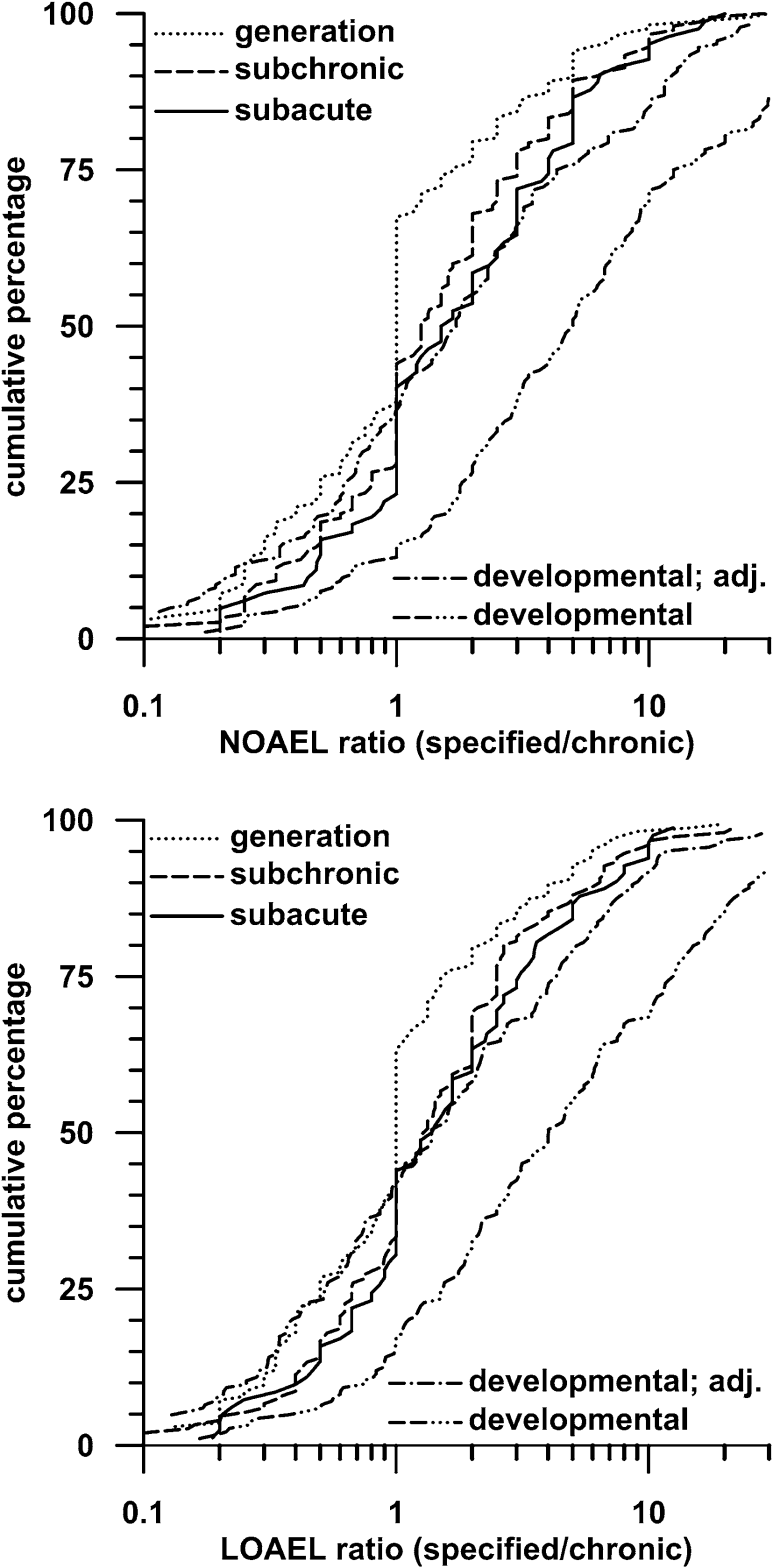
Cumulative distributions of ratios of shorter-term study NOAEL (or LOAEL) to chronic study NOAEL (or LOAEL). Only studies with a dose spacing ≤8 were used to minimize the influence of dose spacing on the NOAEL (or LOAEL) ratio distributions. The types of studies labeled on the graphs were those used to calculate the NOAEL (or LOAEL) ratios. For example, the curve labeled “reproductive” is the cumulative distribution of ratios of reproductive study NOAELs (or LOAELs) to chronic study NOAELs (or LOAELs) for all pesticides. Except for distributions involving developmental studies, all ratio distributions were calculated using the applied concentration of the pesticide in the feed (i.e., ppm). In distributions of ratios of developmental NOAELs (or LOAELs) to chronic study NOAELs (or LOAELs), the NOAELs (or LOAELs) were given as doses (i.e., mg/kg bw per day), because the dose in developmental toxicity studies is usually applied by gavage as a constant dose. In the curves labeled “developmental; adj.”, the chronic NOAELs (or LOAELs) were additionally adjusted to the dose that the animals received at the beginning of the study to account for the dose decrement in feeding studies. For further explanation of this step, see “Study design factors affecting NOAEL ratio distribution”

The influence of exposure duration on NOAELs (or LOAELs) was investigated by calculating the ratios of NOAELs (or LOAELs) from developmental (parental), subacute, subchronic or reproductive (parental) studies to NOAELs (or LOAELs) from chronic studies. Cumulative NOAEL and LOAEL distributions for the single ratios are shown in Fig. 2. Geometric means and statistical comparisons of the distributions are summarized in Table 1. A ratio of 1 indicates that the NOAEL derived from a shorter-term study (developmental, subacute, subchronic or reproductive study) was equal to the NOAEL of the corresponding chronic study. Ratios less than 1 indicate that the shorter-term study NOAEL was lower, whereas ratios greater than 1 indicate that the shorter-term study NOAEL was higher.

**Table 1:** Geometric means of distributions of ratios of NOAELs (or LOAELs) from developmental, subacute, subchronic or reproductive studies to NOAELs (or LOAELs) from chronic studies

NOAEL (or LOAEL) ratio distributions	Dose spacing subset^a^	*n* ^b^	Geometric means of distributions						95th percentile	
			NOAEL			LOAEL			NOAEL	LOAEL
			All	+	−	All	+	−	All	All
Developmental to adjusted chronic	CS	257 (25)	2.5	2.2	4.1	1.7	1.5*	2.9	38.8	25.2
	CS8	184 (19)	1.7	1.6	2.0	1.5	1.4	1.8	18.1	11.9
	CS5	143 (19)	1.4	1.4	1.4	1.3	1.3	1.4	14.0	10.9
Subacute to chronic	CS	129 (26)	2.1	2.1	2.2	1.5	1.5	1.7	21.5	10.0
	CS8	82 (22)	1.8	1.7	2.1	1.6	1.5	2.0	10.0	10.0
	CS5	66 (23)	1.8	1.7	2.2	1.7	1.6	2.1	10.0	10.0
Subchronic to chronic	CS	253 (28)	1.7	1.8	1.5	1.4	1.5	1.3	12.5	10.0
	CS8	150 (21)	1.4	1.5	1.2	1.4	1.5	1.2	10.0	8.5
	CS5	124 (23)	1.4	1.5	1.2	1.4	1.4	1.2	9.7	7.9
Reproductive to chronic	CS	267 (27)	1.2^§^	1.2	1.0	1.1^§^	1.1	1.1	10.0	7.4
	CS8	166 (20)	1.0^§^	1.0	1.1	1.0^$^	1.0	1.0	6.4	6.0
	CS5	128 (20)	1.0^$^	0.9	1.2	1.0^£^	1.0	1.1	5.0	5.3

Two types of statistical comparisons were performed: (1) for a given NOAEL ratio distribution (e.g., subacute to chronic) at given dose spacing cut-off level (e.g., CS8), the three distributions [all compounds (all), compounds with ARfD (+), compounds without ARfD (−)] were analyzed for statistically significant differences; (2) all NOAEL ratio distributions (developmental to chronic, subacute to chronic, subchronic to chronic, reproductive to chronic) of a given dose spacing cut-off level (e.g., CS8) were analyzed for statistically significant differences within the subsets “all compounds (all)”, “compounds with ARfD (+)” and “compounds without ARfD (−)”

^a^CS, CS8 and CS5 represent the whole data set, data set comprising studies with dose spacing ≤8 and data set comprising studies with dose spacing ≤5, respectively

^b^Number of compounds; percentage of compounds without an ARfD given in parentheses

* LOAEL ratio distribution of compounds with an ARfD statistically significantly (Mann–Whitney *U* test, *p* < 0.05) lower than LOAEL ratio distribution of compounds without an ARfD

^§, $, £^ Reproductive to chronic NOAEL or LOAEL ratio distributions of the same dose spacing cut-off level statistically significantly (Kruskal–Wallis test, *p* < 0.05) different from following distributions: §, different from all other distributions; $, different from developmental to chronic and subacute to chronic distributions; £, different from subacute to chronic distribution

Considering only studies with low dose spacing (≤5 or ≤8), NOAEL and LOAEL ratio distributions cover a wide range of values, with 95th percentiles being close to 10. When the NOAELs are expressed as ppm in diet or adjusted for dose decrement, all ratios are closely distributed around 1, meaning that effect levels are similar for chronic and shorter-than-chronic exposure durations. Furthermore, the various NOAEL, respectively, LOAEL distributions are in most cases not statistically significantly different from each other. This reveals that the investigated compounds exert a comparable toxic potency in the different included studies. This was consistently observed in previous analyses of subacute and subchronic NOAELs (and LOAELs) (Zarn et al. 2011). The present analyses show that this can be confirmed for an even wider range of exposure durations including developmental and reproductive toxicity studies. For reproductive toxicity studies, two-thirds of the parental NOAELs and LOAELs were equal to or even lower than the corresponding values from chronic studies.

As noted above, NOAELs were whenever possible as concentrations in feed (e.g., ppm) to avoid biased NOAEL ratio calculations, since the administered dose in feeding studies progressively decreases, because the animals’ intake of feed per body weight steadily decreases as the weight of the animals increases with age (Zarn et al. 2013). Thus, starting with the same administered doses at the beginning of a subacute and a chronic study would show a significantly lower final dose in the chronic study compared to the subacute study, because the feed intake per body weight of rats steadily decreases in the course of the study. Usually, only the mean dose over the whole study duration is reported. Therefore, if the NOAEL of the subacute study is the same as the NOAEL of the chronic study (say 100 ppm), the calculated doses are different because of this dose decrement.

However, NOAELs cannot be expressed as concentrations in feed (e.g., ppm) for developmental NOAELs, because developmental toxicity studies are gavage studies using fixed doses, whereas chronic toxicity studies are typically feeding studies using fixed feed concentrations that yield decreasing doses as the study progresses. For that reason, the developmental to chronic ratios were calculated by expressing the NOAELs as doses (in mg/kg bw per day).The cumulative distribution of the developmental to chronic ratio centers well above 1, which suggests substantial differences between developmental and chronic NOAELs. This difference is due to the fact that chronic NOAELs reported as doses reflect mean intakes averaged over the entire study. Actually, constant pesticide concentrations were administered, meaning that young animals at the beginning of dosing are exposed to an approximately 2.9-fold higher dose compared with the reported mean dose in chronic studies (Zarn et al. 2013). A meaningful analysis of differences between NOAELs from developmental (2 weeks) and chronic (104 weeks) studies therefore requires a comparison of initial doses. When chronic NOAELs are expressed as initial doses at the beginning of the study by correcting the reported values for dose decrement [see above and Zarn et al. (2010, 2011)], the difference between developmental and chronic NOAELs virtually disappears (see Fig. 2).

### Distributions of NOAEL and LOAEL ratios for compounds with and without an ARfD

To investigate differences between acutely toxic and not acutely toxic compounds we distinguished distributions of compounds with and without ARfD and statistically analyzed significant differences between the NOAEL (or LOAEL) ratio distributions by means of pairwise comparisons using the Mann–Whitney *U* test, as summarized in Table 1. In general, the geometric means of ratios derived from studies with low dose spacing (≤5 and ≤8) lie between 1 and 2.2. Even considering the whole CS, geometric means remain ≤2.2 for most of the ratio distributions. Only the developmental to chronic NOAEL ratio distribution shows a higher geometric mean (4.1). Furthermore, the pairwise comparison of ratio distributions showed that there was no statistically significant difference between distributions from compounds with or without an ARfD, the only exception being the developmental to chronic LOAEL ratio, for which the distribution of compounds with an ARfD was significantly different from the distribution of compounds without an ARfD. Overall, NOAELs derived after different exposure durations vary at most by a factor of about 2. As the NOAELs remain unchanged within the included exposure duration range, we conclude that the toxic potency (NOAEL) does not correlate with a prolongation of the exposure duration. Even if the compound is considered not to be acutely toxic and no ARfD was established, effect levels from studies with short-term exposures turned out to be similar to those from chronic studies, which leads us to the conclusion that toxic potencies after chronic and shorter-than-chronic exposures are similar even for not acutely toxic compounds.

### Relationship among ARfD, AOEL and ADI

**Fig. 3:**
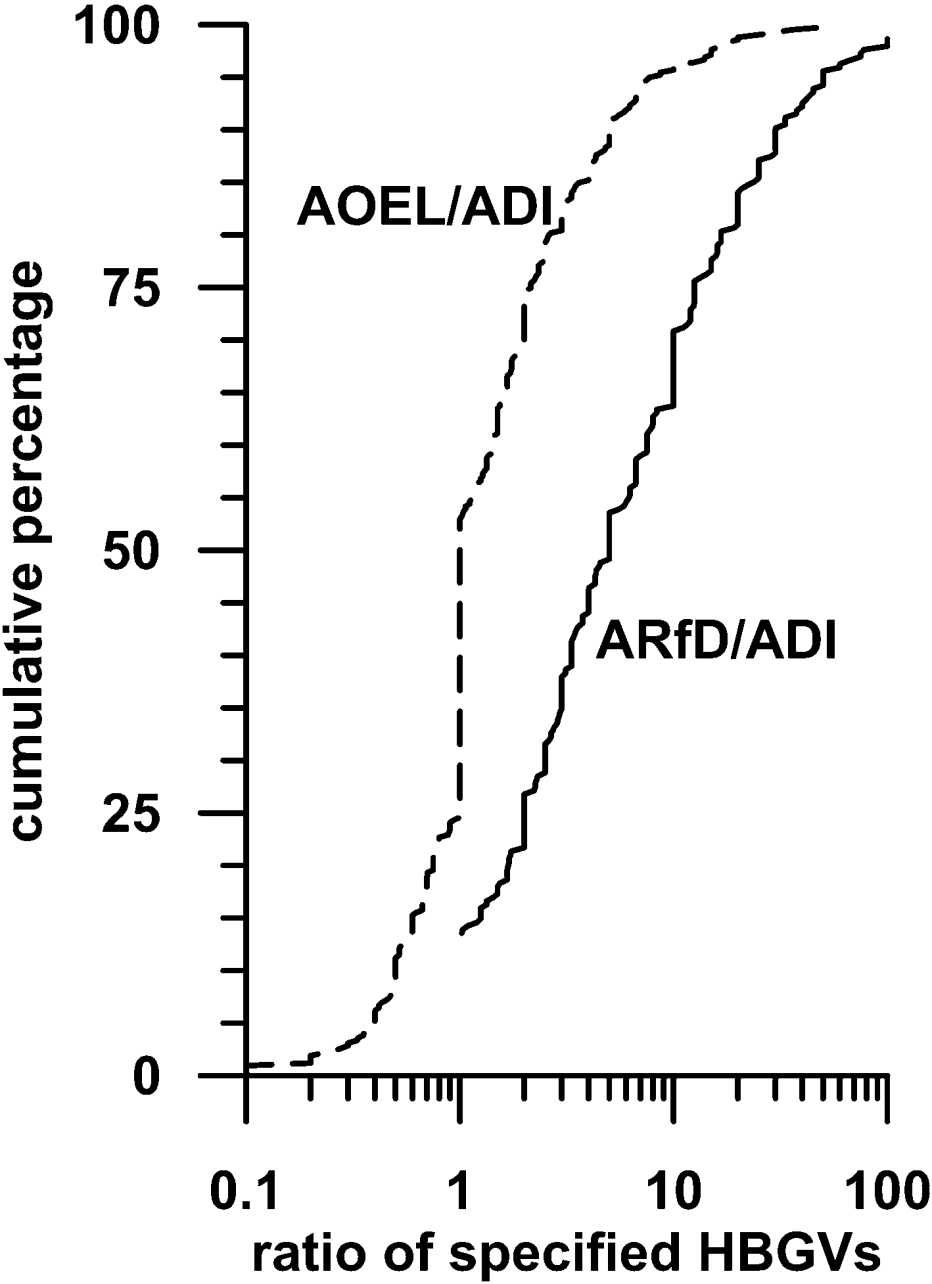
Cumulative distributions of ARfD/ADI ratio (295 compounds) and AOEL/ADI ratio (419 compounds), when AOELs are applied as reported (i.e., if appropriate, being corrected for oral absorption, which applies to 20% of the compounds). The graph of ARfD/ADI ratios starts with approximately 15% at a ratio of 1 because, by definition, an ARfD has to be greater than or equal to the ADI

The ARfD, AOEL and ADI express safe doses for humans following acute, short-term and chronic exposures, respectively, hereby reflecting safe doses (NOAELs) in animal studies with acute, short-term and chronic exposures. To complement our analyses performed on NOAEL and LOAEL distributions gained from animal studies, we compared safe acute (ARfD), short-term (AOEL) and chronic (ADI) human doses by calculating ratios of ARfD to ADI (for the 295 compounds with an ARfD) and ratios of AOEL to ADI (for the 419 compounds with both an ADI and an AOEL). The cumulative distributions are shown in Fig. 3.

The median value for the AOEL/ADI ratio pairs was 1, whereas the 95th percentile was approximately 8. This illustrates that NOAELs driving the AOEL (typically from subchronic studies, as recommended by guidelines) and NOAELs driving the ADI (typically from chronic studies) are similar. However, the AOEL for a pesticide may be lower than the ADI. This is due to the fact that AOELs are expressed as systemic doses, whereas ADIs are external doses. Hence, AOELs occasionally are corrected for oral absorptions less than 80%. Roughly 20% of the compounds fulfil this criterion. As ratio calculations were performed with AOELs as they are reported, lower ratios are obtained for these compounds, so that the distribution depicted in Fig. 3 is shifted to the left. The ratio distributions show nevertheless, that the relationship between the AOEL and the ADI (i.e. safe short-term and chronic doses for humans) is completely consistent with the lack of difference between short-term and chronic NOAELs (i.e., safe doses in experimental animals).

The median value for the corresponding ARfD/ADI ratios was 5, whereas 70% of the pairs had a ratio ≤10. The 95th percentile was 50. Overall, the acute toxic potency of approximately 48% of the PPPs (70% of the 68% of pesticides with an ARfD) was at most 10 times their chronic toxic potency. This is astonishing, in light of the fact, that the endpoints on which the ARfD and ADI are usually based on frequently are completely different, but underlines our finding of small margins between safe doses under short-term and chronic conditions.

### Quantitative comparison of acute and chronic exposure models

**Fig. 4:**
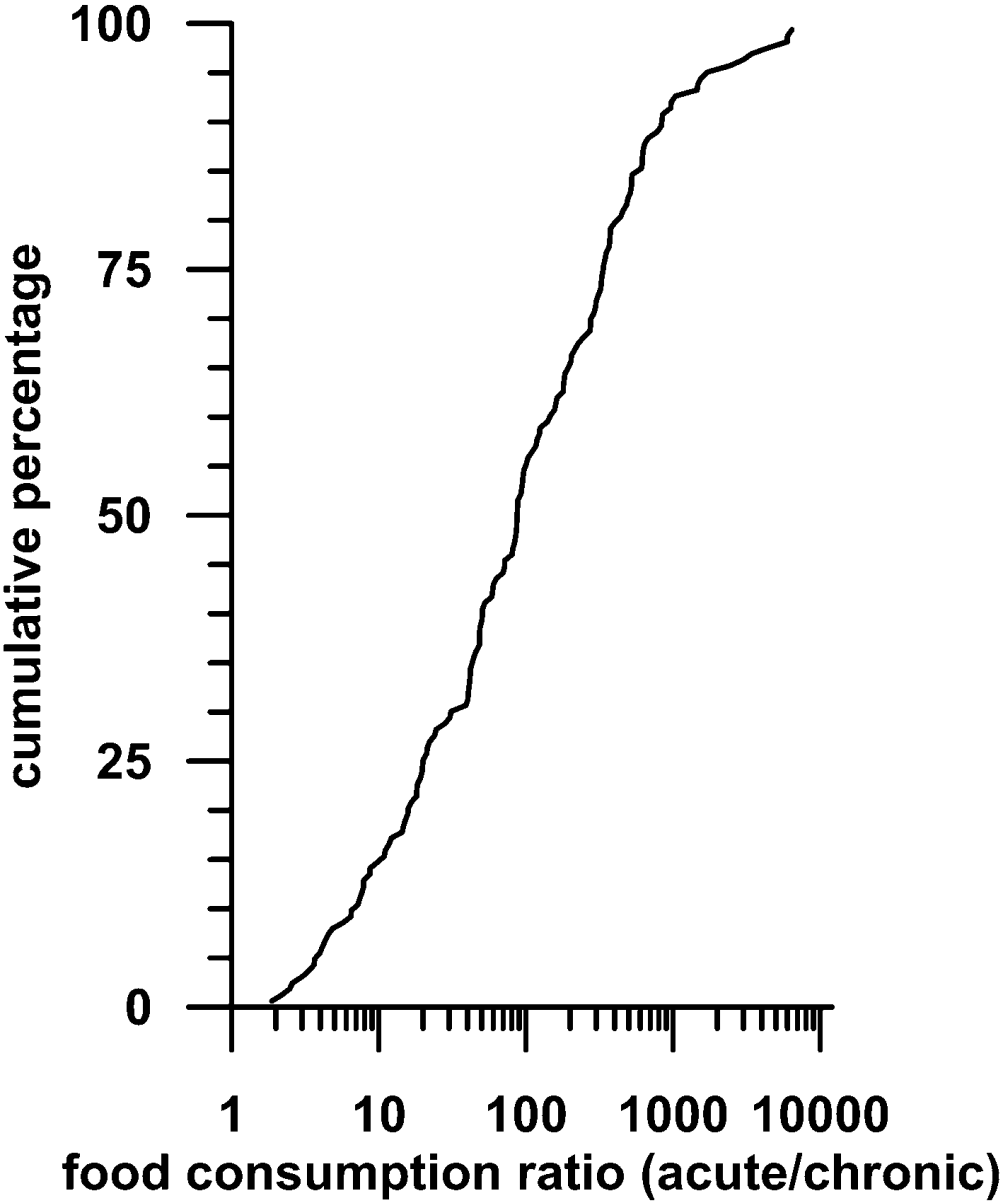
Cumulative distribution of ratios of acute to chronic exposure for 163 food commodities. The chronic (WHO cluster diet E, representing a considerable part of Europe) and acute (highest reported exposure) food consumption data for exposure estimates are from the “PRIMo rev2” exposure model of EFSA (EFSA 2016)

Figure 4 depicts a quantitative comparison of the chronic (WHO cluster diet E, representing a considerable part of Europe) and acute (high percentiles of food consumption surveys) food consumption data used in exposure estimates as provided in revision 2 of the Pesticide Residue Intake Model (PRIMo rev2) of EFSA (EFSA 2016). Consumption data for both acute and chronic exposures were available for 163 food commodities. The median ratio of acute to chronic exposures for the 163 food commodities was 87.3, and the 75th and 95th percentiles were 335.6 and 1708.4, respectively. When it comes to DRA on the basis of residues and MRLs of pesticides in food commodities, these huge differences between acute and chronic food consumption data are in complete disproportion with the virtually non-existing difference between short-term and long-term study NOAELs as well as with the commensurate small difference between ARfDs and ADIs. For example, the chronic assessment considers a peach consumption being approximately 100 fold lower than the acute consumption (see “Discussion”). Accordingly, chronic food consumption for many other commodities is considered to be orders of magnitudes lower than acute food consumptions, whereas exposure duration comparatively has no on the toxic potency (LOAELs).

## Discussion

The present evaluation aims at a statistical analysis of differences in safe doses under different exposure conditions and does not address possible biological causes of differences in NOAELs (or LOAELs). In chronic studies, for example, the histopathological evaluation is performed only after one or 2 years of exposure and hence histopathological effects, which are only observed in the later course of the study, might have been induced as well by late exposures, even if later exposure levels are comparably lower than at the beginning of the study. Therefore, short-term exposures exceeding such HBGVs, which are based on later induced effects, might be of low risk. Certainly, significant and real differences between short-term and long-term NOAELs should be thoroughly evaluated for their biological relevance and their consequences for the DRA in a case-by-case manner. Still, without certainty about the underlying cause of an observed adverse effect, a risk of adverse effects induced by intermittent exposures exceeding the short-term safe dose levels cannot be ruled out.

Furthermore, the present evaluation is focussed on single compound DRAs and does not take into account possibly occurring multiple exposure scenarios in real life. Such scenarios might be of special concern, if an intermittent high exposure above the ADI is identified for a pesticide and is accompanied by exposures to other compounds, which might induce additional stress by similar or different modes of action. Currently, projects are planned to address the biological significance of co-exposures to pesticides, food additives and substances from commercial products (Tsatsakis et al. 2017). Although the present investigation does not take into account multiple exposures scenarios, being conscious about comparable absolute safe dose levels after different exposure durations can nevertheless provide a valuable input to cumulate risk considerations in the future.

### Are risks from intermittent exposures sufficiently covered be the current two-tiered DRA process?

The present investigations were conducted to complement previous analyses on subacute, subchronic and chronic NOAELs by including two further study types, developmental and reproductive toxicity studies, both mandatory parts of the submitted pesticide approval data package, and to promote further discussions on the current DRA assessment, when it comes to intermittent occurring exposures, which exceed chronic exposure estimates. The analyses described in this paper were performed taking into consideration study design factors that can have an impact on NOAEL and LOAEL ratio distributions. Dose spacing was accounted for by assigning subsets meeting a maximum dose spacing of 5 (CS5) or 8 (CS8). For the comparison of developmental and chronic NOAELs (or LOAELs) we considered distorting effects of dose decrement by correcting the reported NOAEL (or LOAEL) dose of the chronic study by an average dose decrement factor of 2.9 (Zarn et al. 2013), which allows a more meaningful comparison of initial doses.

A comparison of NOAELs and LOAELs (expressed as ppm in the diet) identified in studies of different exposure durations showed that the duration of the administration had no significant influence on the derived effect levels. The geometric mean of most of the ratio distributions comparing shorter-than-chronic to chronic NOAEL (or LOAEL) clustered close to 1. Taking the two study types with the greatest difference in exposure duration—namely, the developmental toxicity study (2 weeks) and the chronic toxicity study (104 weeks)—the geometric mean values still differed by a factor of only 2.5, when dose decrement is not accounted for. Considering dose decrement by adjusting the reported averaged doses to actually administered initial doses reveals virtually equally high toxic potencies of pesticides after an exposure duration of 2 weeks compared to an exposure duration of 104 weeks. Furthermore, comparison of compounds with and without an ARfD revealed that the ratios of shorter-than-chronic to chronic NOAELs did not show statistically significantly different distributions. Likewise, most distributions of ratios of shorter-term LOAELs to chronic LOAELs were not statistically significantly different, the only exception being the developmental to chronic LOAEL ratio distribution.

This conclusion is further supported by a comparison of derived HBGVs, which indicates that AOELs are similar to ADIs. Furthermore, approximately 70% of ARfDs are less than tenfold higher than their corresponding ADIs indicating that even the acute toxic potency is not much different from toxic potency following a chronic exposure.

The analyses clearly reveal comparable toxic potencies after short-term and chronic exposure durations, even for not acutely toxic compounds without an ARfD. The current two-tiered DRA process (FAO/WHO 2009), which is based on substantially different acute and chronic food intake models, applies acute and chronic exposure estimates with a much higher margin than the acute and chronic toxic potencies. Essentially, consistent toxic potencies within a broad range of different exposure durations are not reflected by the exposure models applied in the DRA.

Still risks associated with residues of not acutely toxic compounds are only assessed based on very low chronic exposure estimates. Risks emerging from repeated exposures to considerably higher exposure levels are only assessed in the case of acutely toxic compounds. This means that compounds without an ARfD and compounds with significantly different ADI and ARfD bear an unassessed risk regarding short-term exposures to higher exposure levels, because our analyses shows that their chronic NOAELs are not significantly different from their NOAELs at shorter exposure durations. However, the DRA procedure for chronic exposures was neither intended nor developed to account for risks associated with intermittent short-term exposures to levels exceeding the lifetime average exposure level used for chronic assessments. Thus, an exposure model for short-term exposure scenarios involving high exposure levels needs to be integrated into dietary risk assessment, as recommended by the 2015 JMPR (FAO/WHO 2015).

### An illustrative example: bifenazate MRL in peaches

The EU has established an ADI of 0.01 mg/kg bw per day for bifenazate and an ARfD of 0.1 mg/kg bw (EFSA 2017). The MRL for peaches is 2 mg/kg. Toxicologically, the NOAELs (and LOAELs) in mice, rats and dogs are similar over a broad range of exposure durations (Table 2). This indicates that bifenazate has a similar potency over a broad range of exposure durations, as the toxic dose levels derived after a few exposures and after chronic exposures are similar. However, current DRA compares the ADI only to averaged chronic consumer exposures but not to higher intermittent consumer exposures, despite the similar toxic potency after short-term exposures.

**Table 2:** NOAELs and LOAELs for bifenazate identified in studies on rats, mice and dogs

Species	Study	Duration (weeks)	Application route^a^	NOAEL		LOAEL	
				ppm^b^	mg/kg bw	ppm^b^	mg/kg bw
Rat	Developmental	2	Gavage	na^c^	10	na^c^	100
Rat	Subacute	4	Feed	–^d^	–^d^	500	33.3
Rat	Subchronic	13	Feed	40	2.7	200	13.8
Rat	Reproductive	18	Feed	20	1.4	80	5.8
Rat	Chronic	104	Feed	20	1	80	3.9
Mouse	Subacute	4	Feed	–^d^	–^d^	200	33.9
Mouse	Subchronic	13	Feed	50	8	100	16.2
Mouse	Chronic	78	Feed	10	1.5	100	15.4
Dog	Subacute	4	Feed	–^d^	–^d^	300	7.3
Dog	Subchronic	13	Feed	40	0.9	400	10.4
Dog	Chronic	52	Feed	40	1	400	8.9

^a^In the gavage study, the dose was kept constant. In the feeding studies, the concentration in feed was kept constant

^b^Parts per million (equal to mg/kg feed)

^c^Not applicable

^d^The lowest dose was toxic; therefore, no NOAEL was identified

According to the WHO food consumption cluster E diet (representing Central Europe), as given in the “PRIMo rev2” exposure model of EFSA (2015), the chronic peach consumption for a 16.15 kg child is estimated to be 2.2 g/day. One peach on average weighs 127.6 g, and therefore a day’s portion of 2.2 g in the IEDI model is only 1.7% of a single peach. The resulting chronic exposure estimate is 2.7% of the ADI (IEDI calculated by the “PRIMo rev2” exposure model). The regulatory conclusion here is that the MRL of 2 mg/kg for bifenazate in peaches is safe, because the chronic daily intake of peaches—i.e. 1.7% of a single peach assigned an MRL of 2 mg/kg bifenazate—is of no health concern. However, it seems reasonable to assume that peach fanciers may eat one or two peaches occasionally (e.g., during the harvest season). This might lead to intermittent consumption of 100–300 g of peach on single days, which is 45- to 135-fold higher than the average. For bifenazate, actual exposures occurring in such situations are only assessed by comparing actual exposures to the ARfD which is tenfold higher than the ADI. The risks of repeated intermittent exposures, which were shown to have an equally high toxic potency like chronic exposures, remain unassessed even if the exposure is higher than the lifetime averaged chronic exposure estimate.

Here we apply the “PRIMo rev2” exposure model of EFSA (EFSA 2016) to calculate potential actual exposures to bifenazate at the MRL of 2 mg/kg for a child of 16.15 kg. The assumption of a short-term maximum peach intake of 192.6 g within 1 day, which corresponds to 1.5 peaches, results in an actual exposure estimate of 1187% of the ADI. We consider as crucial the fact that the ADI is based on a NOAEL that is not significantly different from all other NOAELs for this compound that were derived after shorter-term exposures. In view of the similar toxic potency of this pesticide after short-term exposures, occasional repeated exceedances of the ADI may be critical, but currently remain unassessed for pesticides without an ARfD or for pesticides with ARfDs significantly higher than their ADIs.

## Conclusion

There is a need to develop DRA methodologies that take into account equally high toxic potencies of pesticides at different exposure durations.

## Electronic supplementary material

Below is the link to the electronic supplementary material.

Supplementary material 1 (DOCX 41 kb)

## Abbreviations

ADI: Acceptable daily intake
AOEL: Acceptable operator exposure level
ARfD: Acute reference dose
bw: Body weight
CS/CS5/CS8: Complete data set of studies/subset of studies with dose spacing ≤5/subset of studies with dose spacing ≤8
DRA: Dietary risk assessment
EFSA: European Food Safety Authority
HBGV: Health-based guidance value
IEDI: International estimated daily intake
IESTI: International estimated short-term intake
JMPR: Joint FAO/WHO Meeting on Pesticide Residues
LOAEL: Lowest-observed-adverse-effect level
MRL: Maximum residue level
NOAEL: No-observed-adverse-effect level
PPP: Plant protection product
US EPA: United States Environmental Protection Agency
