# Supplementary material for: Current pesticide dietary risk assessment in light of comparable animal study NOAELs after chronic and short-termed exposure durations
Source: Arch Toxicol. 2017 Sep 19;92(1):157–67. doi: 10.1007/s00204-017-2052-4 (PMC5773667; doi:10.1007/s00204-017-2052-4)
Supplement: Supplementary file 1 — Supplementary material 1 (DOCX 41 kb) [file 204_2017_2052_MOESM1_ESM.docx]

This supplemental data addendum accompanies the publication “Current pesticide dietary risk assessment in light of comparable animal study NOAELs after chronic and short-termed exposure durations” (JA Zarn & CD O’Brien 2017)

**Pesticide database used for current publication**

The Microsoft Office Access 2007 database contains:

- Compound-specific data for 1550 pesticides (for data categories entered see upper part of ***Supplemental Table 1***)
- For 506 out of 1550 pesticides, study-specific data on 7718 toxicity studies (for data categories entered see lower part of ***Supplemental Table 1***)

Study selection for NOAEL (or LOAEL) ratio calculations was as follows:

- If a study was evaluated by more than one authority, preference was given to EU, then to WHO and finally to USA evaluations. Following these selection criteria a total of 6058 studies out of 7718 available studies were considered relevant.
- 3238 out of these 6058 relevant studies were rat studies.
- Out of these 3238 rat studies, only those were selected, in which both a NOAEL and a LOAEL was reported.

This results in 2200 informative rat studies for 436 compounds. The 436 pesticides are listed below (***Supplemental Table 2***).

If more than one study for a given study type (e.g. subacute study) with a given compound was available (quite rare situation), the study with the lowest dose spacing (LOAEL to NOAEL ratio) was selected for NOAEL (or LOAEL) ratio calculations with NOAELs (or LOAELs) from other studies.

The described selection procedures were automated by means of the Access database.

**Quality checks**

Certain automated and manual quality checks were included. For example, automatically and continuously it was checked whether the entered NOAEL and LOAEL are part of the doses applied and whether the conversion factor between the concentration in feed and the reported dose is in the expected range typical for the respective study type.

**Supplemental Table 1: Data categories entered into Access database**

| Data category | Remarks |
| --- | --- |
| **Details on the compound** | |
| Name |  |
| Structure (ChemDraw file) |  |
| CAS Nr |  |
| CAS name |  |
| IUPAC name |  |
| SMILES code |  |
| Chemical class |  |
| Cramer class |  |
| Pesticidal function |  |
| Legal status EU |  |
| ADI | All, from any authority |
| ARfD | All, from any authority |
| AOEL | All, from any authority |
| GHS classification |  |
| **Details on toxicity studies** | |
| Evaluating body | EU, WHO, USA, etc. |
| Year of evaluation |  |
| Study author |  |
| Year study performed |  |
| Species |  |
| Strain |  |
| Animals per dose group |  |
| Exposure duration | weeks |
| Dose levels (all) |  |
| NOAEL | As reported by the evaluating authority. Dose and food concentration (if feeding study) |
| LOAEL | As reported by the evaluating authority. Dose and food concentration (if feeding study) |
| Application method | Capsule, gavage, food, water. Information whether the dose was adjusted for changes in bodyweight. |

**Supplemental Table 2: Compounds used for the current publication**

| name | CAS number | ADI | ARfD | AOEL | primary use | status in EU (end of 2016) |
| --- | --- | --- | --- | --- | --- | --- |
|  |  | mg/kg bw | | |  |  |
| 2,4-D | 94-75-7 | 0.05 | 0.75 | 0.15 | herbicide | approved |
| Acetochlor | 34256-82-1 | 0.0036 | 1.5 | 0.02 | herbicide | not approved |
| Aclonifen | 74070-46-5 | 0.07 |  | 0.07 | herbicide | approved |
| Alachlor | 15972-60-8 |  |  |  | herbicide | not approved |
| Ametryn | 834-12-8 |  |  |  | herbicide | not approved |
| Amicarbazone | 129909-90-6 |  |  |  | herbicide | not approved |
| Amidosulfuron | 120923-37-7 | 0.2 |  | 1.4 | herbicide | approved |
| Aminocyclopyrachlor | 858956-08-8 |  |  |  | herbicide | no information |
| Aminopyralid | 150114-71-9 | 0.26 | 0.26 | 0.26 | herbicide | approved |
| Amitrol | 61-82-5 | 0.001 |  | 0.001 | herbicide | not approved |
| Asulam | 3337-71-1 | 0.36 | 1 | 0.46 | herbicide | not approved |
| Atrazin | 1912-24-9 | 0.02 | 0.1 |  | herbicide | not approved |
| Azimsulfuron | 120162-55-2 | 0.1 |  | 0.1 | herbicide | approved |
| Beflubutamid | 113614-08-7 | 0.02 |  | 0.3 | herbicide | approved |
| Benfluralin | 1861-40-1 | 0.005 |  | 0.005 | herbicide | approved |
| Bensulfuron | 99283-01-9 | 0.2 |  | 0.12 | herbicide | approved |
| Bentazon | 25057-89-0 | 0.1 | 0.25 | 0.13 | herbicide | approved |
| Bifenox | 42576-02-3 | 0.3 | 0.5 | 0.125 | herbicide | approved |
| Bispyribac | 125401-75-4 | 0.01 |  | 0.072 | herbicide | approved |
| Bromacil | 314-40-9 |  |  |  | herbicide | not approved |
| Bromoxynil | 1689-84-5 | 0.01 | 0.04 | 0.01 | herbicide | approved |
| Butralin | 33629-47-9 |  |  |  | herbicide | not approved |
| Carbetamid | 16118-49-3 | 0.06 | 0.3 | 0.12 | herbicide | approved |
| Carfentrazone-ethyl | 128639-02-1 | 0.03 |  | 0.6 | herbicide | approved |
| Chloridazon | 1698-60-8 | 0.1 |  | 0.2 | herbicide | approved |
| Chlorotoluron | 15545-48-9 | 0.04 |  | 0.215 | herbicide | approved |
| Chlorpropham | 101-21-3 | 0.05 | 0.5 | 0.05 | herbicide | approved |
| Chlorsulfuron | 64902-72-3 | 0.2 |  | 0.43 | herbicide | approved |
| Chlorthal-dimethyl | 1861-32-1 | 0.01 | 0.5 |  | herbicide | not approved |
| Clethodim | 99129-21-2 | 0.16 |  | 0.2 | herbicide | approved |
| Clodinafop-propargyl | 105512-06-9 | 0.003 | 0.05 | 0.026 | herbicide | approved |
| Clomazone | 81777-89-1 | 0.133 |  | 0.133 | herbicide | approved |
| Clopyralid | 1702-17-6 | 0.15 |  | 1 | herbicide | approved |
| Cloransulam-methyl | 147150-35-4 |  |  |  | herbicide | no information |
| Cyanamid | 420-04-2 | 0.002 | 0.05 | 0.002 | herbicide | not approved |
| Cycloxydim | 101205-02-1 | 0.07 | 2 | 0.1 | herbicide | approved |
| Cyhalofop-butyl | 122008-85-9 | 0.003 |  | 0.03 | herbicide | approved |
| Desmedipham | 13684-56-5 | 0.03 | 0.1 | 0.04 | herbicide | approved |
| Dicamba | 1918-00-9 | 0.3 | 0.3 | 0.3 | herbicide | approved |
| Dichlobenil | 1194-65-6 | 0.01 | 0.45 | 0.01 | herbicide | not approved |
| Dichlorprop-P | 15165-67-0 | 0.06 | 0.5 | 0.35 | herbicide | approved |
| Diclofop-methyl | 51338-27-3 | 0.001 | 0.03 | 0.003 | herbicide | approved |
| Diflufenican | 83164-33-4 | 0.2 |  | 0.11 | herbicide | approved |
| Diflufenzopyr | 109293-97-2 |  |  |  | herbicide | not approved |
| Dimefuron | 34205-21-5 |  |  |  | herbicide | not approved |
| Dimethachlor | 50563-36-5 | 0.1 | 0.5 | 0.1 | herbicide | approved |
| Dimethenamid | 87674-68-8 | 0.02 | 0.25 | 0.04 | herbicide | not approved |
| Diquat | 2764-72-9 | 0.002 |  | 0.001 | herbicide | approved |
| Diuron | 330-54-1 | 0.007 | 0.016 | 0.007 | herbicide | approved |
| Ethalfluralin | 55283-68-6 |  |  |  | herbicide | not approved |
| Ethametsulfuron-methyl | 97780-06-8 |  |  |  | herbicide | pending |
| Fenoxaprop | 95617-09-7 |  |  |  | herbicide | not approved |
| Flazasulfuron | 104040-78-0 | 0.013 |  | 0.02 | herbicide | approved |
| Florasulam | 145701-23-1 | 0.05 |  | 0.05 | herbicide | approved |
| Fluazifop-butyl | 79241-46-6 |  |  |  | herbicide | not approved |
| Flucarbazon | 145026-88-6 |  |  |  | herbicide | not approved |
| Flufenacet | 142459-58-3 | 0.005 | 0.017 | 0.017 | herbicide | approved |
| Flumioxazin | 103361-09-7 | 0.009 | 0.05 | 0.018 | herbicide | approved |
| Fluometuron | 2164-17-2 | 0.0005 | 0.008 | 0.008 | herbicide | approved |
| Flurochloridon | 61213-25-0 | 0.04 | 0.04 | 0.04 | herbicide | approved |
| Fluroxypyr | 69377-81-7 | 0.8 |  | 0.8 | herbicide | approved |
| Fluthiacet | 149253-65-6 |  |  |  | herbicide | no information |
| Fomesafen | 72178-02-0 |  |  |  | herbicide | not approved |
| Foramsulfuron | 173159-57-4 | 0.5 |  | 0.1 | herbicide | approved |
| Glufosinat | 51276-47-2 | 0.021 | 0.021 | 0.0021 | herbicide | approved |
| Glyphosat | 1071-83-6 | 0.3 |  | 0.2 | herbicide | approved |
| Halauxifen-methyl | 943832-60-8 | 0.058 | 0.058 | 0.058 | herbicide | approved |
| Halosulfuron-methyl | 100784-20-1 | 0.063 | 0.5 | 0.063 | herbicide | approved |
| Haloxyfop | 72619-32-0 | 0.00065 | 0.075 |  | herbicide | not approved |
| Imazamox | 114311-32-9 | 9 |  | 14 | herbicide | approved |
| Imazapic | 104098-48-8 | 0.46 |  |  | herbicide | not approved |
| Imazapyr | 81334-34-1 | 2.5 |  |  | herbicide | not approved |
| Imazaquin | 81335-37-7 | 0.25 |  | 0.25 | herbicide | approved |
| Imazosulfuron | 122548-33-8 | 0.75 |  | 0.53 | herbicide | approved |
| Indaziflam | 950782-86-2 |  |  |  | herbicide | no information |
| Iodosulfuron-methyl | 144550-36-7 | 0.03 |  | 0.05 | herbicide | approved |
| Isoproturon | 34123-59-6 | 0.015 |  | 0.015 | herbicide | not approved |
| Isoxaben | 82558-50-7 | 0.05 |  | 0.25 | herbicide | approved |
| Isoxaflutol | 141112-29-0 | 0.02 |  | 0.02 | herbicide | approved |
| Lenacil | 2164-08-1 | 0.12 |  | 0.4 | herbicide | approved |
| MCPA | 94-74-6 | 0.05 | 0.15 | 0.04 | herbicide | approved |
| Mecoprop | 7085-19-0 | 0.01 |  | 0.04 | herbicide | not approved |
| Mesotrione | 104206-82-8 | 0.01 | 0.02 | 0.015 | herbicide | approved |
| Metamitron | 41394-05-2 | 0.03 | 0.1 | 0.036 | herbicide | approved |
| Metazachlor | 67129-08-2 | 0.08 | 0.5 | 0.2 | herbicide | approved |
| Metobromuron | 3060-89-7 | 0.008 | 0.3 | 0.016 | herbicide | approved |
| Metolachlor, S- | 87392-12-9 | 0.1 |  | 0.15 | herbicide | approved |
| Metosulam | 139528-85-1 | 0.05 | 0.25 | 0.02 | herbicide | approved |
| Metribuzin | 21087-64-9 | 0.013 | 0.02 | 0.02 | herbicide | approved |
| Metsulfuron-methyl | 74223-64-6 | 0.22 | 0.25 | 0.25 | herbicide | approved |
| Monolinuron | 1746-81-2 | 0.003 |  |  | herbicide | not approved |
| Napropamid | 15299-99-7 |  |  |  | herbicide | pending |
| Nicosulfuron | 111991-09-4 | 2 |  | 0.8 | herbicide | approved |
| Orthosulfamuron | 213464-77-8 |  |  |  | herbicide | pending |
| Oryzalin | 19044-88-3 | 0.05 |  | 0.05 | herbicide | approved |
| Oxadiazon | 19666-30-9 | 0.0036 | 0.12 | 0.05 | herbicide | approved |
| Oxyfluorfen | 42874-03-3 | 0.003 | 0.3 | 0.013 | herbicide | approved |
| Paraquat | 4685-14-7 | 0.004 | 0.005 | 0.0004 | herbicide | not approved |
| Penoxsulam | 219714-96-2 | 0.05 |  | 0.18 | herbicide | approved |
| Pethoxamid | 106700-29-2 | 0.01 | 0.08 | 0.02 | herbicide | approved |
| Picloram | 1918-02-1 | 0.3 | 0.3 | 0.3 | herbicide | approved |
| Picolinafen | 137641-05-5 | 0.014 | 0.05 | 0.03 | herbicide | approved |
| Pinoxaden | 243973-20-8 | 0.1 | 0.1 | 0.1 | herbicide | approved |
| Propanil | 709-98-8 | 0.02 | 0.07 | 0.02 | herbicide | pending |
| Propaquizafop | 111479-05-1 | 0.015 |  | 0.04 | herbicide | approved |
| Propazin | 139-40-2 |  |  |  | herbicide | not approved |
| Propham | 122-42-9 |  |  |  | herbicide | not approved |
| Propisochlor | 86763-47-5 | 0.025 | 0.05 | 0.025 | herbicide | not approved |
| Propoxycarbazon | 181274-15-7 | 0.4 |  | 0.3 | herbicide | approved |
| Propyzamid | 23950-58-5 | 0.02 |  | 0.08 | herbicide | approved |
| Prosulfocarb | 52888-80-9 | 0.005 | 0.1 | 0.007 | herbicide | approved |
| Prosulfuron | 94125-34-5 | 0.02 |  | 0.06 | herbicide | approved |
| Pyraflufen-ethyl | 129630-17-7 | 0.2 | 0.2 | 0.1 | herbicide | approved |
| Pyrasulfotol | 365400-11-9 |  |  |  | herbicide | not approved |
| Pyridate | 55512-33-9 | 0.036 | 0.4 | 0.036 | herbicide | approved |
| Pyroxsulam | 422556-08-9 | 0.9 |  | 0.7 | herbicide | approved |
| Quinmerac | 90717-03-6 | 0.08 | 0.3 | 0.08 | herbicide | approved |
| Quizalofop-ethyl | 76578-14-8 |  |  |  | herbicide | not approved |
| Quizalofop-P-tefuryl | 119738-06-6 | 0.013 | 0.1 | 0.01 | herbicide | approved |
| Rimsulfuron | 122931-48-0 | 0.1 |  | 0.07 | herbicide | approved |
| Saflufenacil | 372137-35-4 |  |  |  | herbicide | not approved |
| Sulcotrione | 99105-77-8 | 0.0004 |  | 0.0006 | herbicide | approved |
| Sulfentrazon | 122836-35-5 |  |  |  | herbicide | not approved |
| Sulfosulfuron | 141776-32-1 | 0.24 |  | 0.4 | herbicide | approved |
| Tembotrion | 335104-84-2 | 0.0004 | 0.1 | 0.0007 | herbicide | approved |
| Terbuthylazin | 5915-41-3 | 0.004 | 0.008 | 0.0032 | herbicide | approved |
| Thiazopyr | 117718-60-2 |  |  |  | herbicide | not approved |
| Thiencarbazon-methyl | 317815-83-1 | 0.23 |  | 0.12 | herbicide | approved |
| Thiobencarb | 28249-77-6 |  |  |  | herbicide | not approved |
| Topramezon | 210631-68-8 | 0.001 | 0.001 | 0.0006 | herbicide | pending |
| Tralkoxydim | 87820-88-0 | 0.005 | 0.01 | 0.005 | herbicide | approved |
| Tri-allat | 2303-17-5 | 0.025 | 0.6 | 0.032 | herbicide | approved |
| Triasulfuron | 82097-50-5 | 0.01 |  | 0.3 | herbicide | not approved |
| Tribenuron-methyl | 101200-48-0 | 0.01 | 0.2 | 0.07 | herbicide | approved |
| Triclopyr | 55335-06-3 | 0.03 | 0.3 | 0.05 | herbicide | approved |
| Trifluralin | 1582-09-8 | 0.015 |  | 0.026 | herbicide | not approved |
| Triflusulfuron-methyl | 126535-15-7 | 0.04 | 1.2 | 0.04 | herbicide | approved |
| Tritosulfuron | 142469-14-5 | 0.06 |  | 0.15 | herbicide | approved |
| Acibenzolar-S-methyl | 135158-54-2 | 0.03 | 0.03 | 0.03 | fungicide | approved |
| Amisulbrom | 348635-87-0 | 0.1 | 0.3 | 0.15 | fungicide | approved |
| Anilazin | 101-05-3 | 0.1 |  |  | fungicide | not approved |
| Azaconazol | 60207-31-0 |  |  |  | fungicide | not approved |
| Azoxystrobin | 131860-33-8 | 0.2 |  | 0.2 | fungicide | approved |
| Benalaxyl | 71626-11-4 | 0.04 |  | 0.06 | fungicide | approved |
| Benthiavalicarb-isopropyl | 177406-68-7 | 0.1 |  | 5 | fungicide | approved |
| Benzovindiflupyr | 1072957-71-1 | 0.05 | 0.1 | 0.04 | fungicide | approved |
| Bitertanol | 55179-31-2 | 0.003 | 0.01 | 0.01 | fungicide | not approved |
| Bixafen | 581809-46-3 | 0.02 | 0.2 | 0.13 | fungicide | approved |
| Boscalid | 188425-85-6 | 0.04 |  | 0.1 | fungicide | approved |
| Bromuconazol | 116255-48-2 | 0.01 | 0.1 | 0.025 | fungicide | approved |
| Bupirimate | 41483-43-6 | 0.05 |  | 0.05 | fungicide | approved |
| Captan | 133-06-2 | 0.1 | 0.3 | 0.1 | fungicide | approved |
| Carbendazim | 10605-21-7 | 0.02 | 0.02 | 0.02 | fungicide | not approved |
| Carboxin | 5234-68-4 | 0.008 |  | 0.055 | fungicide | approved |
| Chloropicrin | 76-06-2 | 0.001 | 0.001 | 0.001 | fungicide | not approved |
| Chlorothalonil | 1897-45-6 | 0.015 | 0.6 | 0.009 | fungicide | approved |
| Cyazofamid | 120116-88-3 | 0.17 |  | 0.3 | fungicide | approved |
| Cyflufenamid | 180409-60-3 | 0.04 | 0.05 | 0.03 | fungicide | approved |
| Cymoxanil | 57966-95-7 | 0.013 | 0.08 | 0.01 | fungicide | approved |
| Cyproconazol | 94361-06-5 | 0.02 | 0.02 | 0.02 | fungicide | approved |
| Cyprodinil | 121552-61-2 | 0.03 |  | 0.03 | fungicide | approved |
| Dazomet | 533-74-4 | 0.01 | 0.03 | 0.015 | fungicide | approved |
| Dicloran | 99-30-9 | 0.005 | 0.025 | 0.005 | fungicide | not approved |
| Diethofencarb | 87130-20-9 | 0.43 |  | 0.5 | fungicide | approved |
| Difenoconazol | 119446-68-3 | 0.01 | 0.16 | 0.16 | fungicide | approved |
| Dimethomorph | 110488-70-5 | 0.05 | 0.6 | 0.15 | fungicide | approved |
| Dimoxystrobin | 149961-52-4 | 0.004 | 0.004 | 0.02 | fungicide | approved |
| Dinocap | 39300-45-3 | 0.004 | 0.004 | 0.003 | fungicide | not approved |
| Diphenylamin | 122-39-4 | 0.075 |  | 0.1 | fungicide | not approved |
| Dithianon | 3347-22-6 | 0.01 | 0.12 | 0.0135 | fungicide | approved |
| Dodemorph | 1593-77-7 | 0.082 | 0.33 | 0.033 | fungicide | approved |
| Dodin | 2439-10-3 | 0.1 | 0.1 | 0.045 | fungicide | approved |
| Epoxiconazol | 133855-98-8 | 0.008 | 0.023 | 0.008 | fungicide | approved |
| Ethaboxam | 162650-77-3 |  |  |  | fungicide | not approved |
| Ethoxyquin | 91-53-2 |  |  |  | fungicide | not approved |
| Etridiazol | 2593-15-9 | 0.015 | 0.15 | 0.03 | fungicide | approved |
| Famoxadon | 131807-57-3 | 0.012 | 0.2 | 0.0048 | fungicide | approved |
| Fenamidon | 161326-34-7 | 0.03 |  | 0.3 | fungicide | approved |
| Fenarimol | 60168-88-9 | 0.01 | 0.02 |  | fungicide | not approved |
| Fenbuconazol | 114369-43-6 | 0.006 | 0.3 | 0.02 | fungicide | approved |
| Fenhexamid | 126833-17-8 | 0.2 |  | 0.2 | fungicide | approved |
| Fenpiclonil | 74738-17-3 |  |  |  | fungicide | not approved |
| Fenpropidin | 67306-00-7 | 0.02 | 0.02 | 0.02 | fungicide | approved |
| Fenpropimorph | 67564-91-4 | 0.003 | 0.03 | 0.007 | fungicide | approved |
| Fenpyrazamine | 473798-59-3 | 0.13 | 0.3 | 0.2 | fungicide | approved |
| Ferbam | 14484-64-1 | 0.003 |  |  | fungicide | not approved |
| Fluazinam | 79622-59-6 | 0.01 | 0.07 | 0.004 | fungicide | approved |
| Fludioxonil | 131341-86-1 | 0.37 |  | 0.59 | fungicide | approved |
| Fluopicolid | 239110-15-7 | 0.08 | 0.18 | 0.05 | fungicide | approved |
| Fluopyram | 658066-35-4 | 0.012 | 0.5 | 0.05 | fungicide | approved |
| Fluoxastrobin | 361377-29-9 | 0.015 | 0.3 | 0.03 | fungicide | approved |
| Fluquinconazol | 136426-54-5 | 0.002 | 0.02 | 0.001 | fungicide | approved |
| Flusilazol | 85509-19-9 | 0.002 | 0.005 | 0.005 | fungicide | not approved |
| Flutianil | 958647-10-4 |  |  |  | fungicide | no information |
| Flutolanil | 66332-96-5 | 0.09 |  | 0.56 | fungicide | approved |
| Flutriafol | 76674-21-0 | 0.01 | 0.05 | 0.05 | fungicide | approved |
| Fluxapyroxad | 907204-31-3 | 0.02 | 0.25 | 0.04 | fungicide | approved |
| Folpet | 133-07-3 | 0.1 | 0.2 | 0.1 | fungicide | approved |
| Fosetyl-Aluminium | 39148-24-8 | 3 |  | 5 | fungicide | approved |
| Fuberidazol | 3878-19-1 | 0.0072 | 0.08 | 0.0072 | fungicide | approved |
| Furfural | 98-01-1 |  |  |  | fungicide | not approved |
| Guazatine | 108173-90-6 | 0.0048 | 0.04 | 0.002 | fungicide | not approved |
| Hexaconazol | 79983-71-4 | 0.005 |  |  | fungicide | not approved |
| Hymexazol | 10004-44-1 | 0.17 | 0.5 | 0.17 | fungicide | approved |
| Imazalil | 35554-44-0 | 0.025 | 0.05 | 0.05 | fungicide | approved |
| Ipconazol | 125225-28-7 | 0.015 | 0.015 | 0.015 | fungicide | approved |
| Iprodion | 36734-19-7 | 0.06 |  | 0.3 | fungicide | approved |
| Iprovalicarb | 140923-17-7 | 0.015 |  | 0.015 | fungicide | approved |
| Isopyrazam | 881685-58-1 | 0.03 | 0.2 | 0.05 | fungicide | approved |
| Kresoxim-methyl | 143390-89-0 | 0.4 |  | 0.9 | fungicide | approved |
| Mancozeb | 8018-01-7 | 0.05 | 0.6 | 0.035 | fungicide | approved |
| Mandestrobin | 173662-97-0 | 0.19 |  | 0.19 | fungicide | approved |
| Mandipropamid | 374726-62-2 | 0.15 |  | 0.17 | fungicide | approved |
| Maneb | 12427-38-2 | 0.05 | 0.2 | 0.03 | fungicide | not approved |
| Meptyldinocap | 131-72-6 | 0.016 | 0.12 | 0.008 | fungicide | approved |
| Metalaxyl | 57837-19-1 | 0.08 | 0.5 | 0.08 | fungicide | approved |
| Metam | 144-54-7 | 0.001 | 0.1 | 0.001 | fungicide | approved |
| Metconazol | 125116-23-6 | 0.01 | 0.01 | 0.01 | fungicide | approved |
| Metiram | 9006-42-2 | 0.03 |  | 0.016 | fungicide | approved |
| Metrafenon | 220899-03-6 | 0.25 |  | 0.43 | fungicide | approved |
| Myclobutanil | 88671-89-0 | 0.025 | 0.31 | 0.03 | fungicide | approved |
| Orthophenylphenol | 90-43-7 | 0.4 |  | 0.4 | fungicide | approved |
| Hydroxyquinoline, 8- | 148-24-3 | 0.05 | 0.05 | 0.05 | fungicide | approved |
| Penconazol | 66246-88-6 | 0.03 | 0.5 | 0.03 | fungicide | approved |
| Pencycuron | 66063-05-6 | 0.2 |  | 0.15 | fungicide | approved |
| Penflufen | 494793-67-8 | 0.04 | 0.5 | 0.077 | fungicide | approved |
| Penthiopyrad | 183675-82-3 | 0.1 | 0.75 | 0.1 | fungicide | approved |
| Picoxystrobin | 117428-22-5 | 0.043 |  | 0.043 | fungicide | approved |
| Prochloraz | 67747-09-5 | 0.01 | 0.025 | 0.02 | fungicide | approved |
| Procymidon | 32809-16-8 | 0.0028 | 0.012 | 0.012 | fungicide | not approved |
| Propamocarb | 24579-73-5 | 0.29 | 1 | 0.29 | fungicide | approved |
| Propiconazol | 60207-90-1 | 0.04 | 0.3 | 0.1 | fungicide | approved |
| Propineb | 12071-83-9 | 0.007 | 0.1 | 0.003 | fungicide | approved |
| Proquinazid | 189278-12-4 | 0.01 | 0.2 | 0.02 | fungicide | approved |
| Prothioconazol | 178928-70-6 | 0.01 | 0.01 | 0.2 | fungicide | approved |
| Prothioconazol, desthio | 120983-64-4 |  |  |  | fungicide | no information |
| Pyraclostrobin | 175013-18-0 | 0.03 | 0.03 | 0.015 | fungicide | approved |
| Pyrazophos | 13457-18-6 | 0.004 |  |  | fungicide | not approved |
| Pyrimethanil | 53112-28-0 | 0.17 |  | 0.12 | fungicide | approved |
| Pyriofenon | 688046-61-9 | 0.07 |  | 0.15 | fungicide | approved |
| Quinoxyfen | 124495-18-7 | 0.2 |  | 0.14 | fungicide | approved |
| Quintozen | 82-68-8 | 0.01 |  |  | fungicide | not approved |
| Sedaxan | 874967-67-6 | 0.11 | 0.3 | 0.28 | fungicide | approved |
| Spiroxamine | 118134-30-8 | 0.025 | 0.1 | 0.015 | fungicide | approved |
| Tebuconazol | 107534-96-3 | 0.03 | 0.03 | 0.03 | fungicide | approved |
| Tecnazen | 117-18-0 | 0.02 |  |  | fungicide | not approved |
| Tetraconazol | 112281-77-3 | 0.004 | 0.05 | 0.03 | fungicide | approved |
| Thiabendazol | 148-79-8 | 0.1 |  | 0.1 | fungicide | approved |
| Thiophanat-methyl | 23564-05-8 | 0.08 | 0.2 | 0.08 | fungicide | approved |
| Thiram | 137-26-8 | 0.01 | 0.6 | 0.02 | fungicide | approved |
| Tolclofos-methyl | 57018-04-9 | 0.064 |  | 0.2 | fungicide | approved |
| Tolylfluanid | 731-27-1 | 0.1 | 0.25 | 0.3 | fungicide | not approved |
| Triadimefon | 43121-43-3 | 0.03 | 0.08 |  | fungicide | not approved |
| Triadimenol | 55219-65-3 | 0.05 | 0.05 | 0.05 | fungicide | approved |
| Triazoxid | 72459-58-6 | 0.0002 | 0.015 | 0.001 | fungicide | approved |
| Tricyclazol | 41814-78-2 |  |  |  | fungicide | not approved |
| Trifloxystrobin | 141517-21-7 | 0.1 |  | 0.06 | fungicide | approved |
| Triflumizole | 68694-11-1 | 0.05 | 0.1 | 0.05 | fungicide | approved |
| Triforin | 26644-46-2 | 0.02 |  |  | fungicide | not approved |
| Triticonazol | 131983-72-7 | 0.025 | 0.05 | 0.025 | fungicide | approved |
| Valifenalat | 283159-90-0 | 0.07 |  | 0.07 | fungicide | approved |
| Vinclozolin | 50471-44-8 | 0.005 | 0.06 |  | fungicide | not approved |
| Zineb | 12122-67-7 | 0.03 |  |  | fungicide | not approved |
| Ziram | 137-30-4 | 0.006 | 0.08 | 0.015 | fungicide | approved |
| Abamectin | 71751-41-2 | 0.0025 | 0.005 | 0.0025 | insecticide | approved |
| Acephat | 30560-19-1 |  |  |  | insecticide | not approved |
| Acetamiprid | 135410-20-7 | 0.07 | 0.1 | 0.07 | insecticide | approved |
| Aldicarb | 116-06-3 |  |  |  | insecticide | not approved |
| Amitraz | 33089-61-1 | 0.003 | 0.01 |  | insecticide | not approved |
| Azadirachtin | 11141-17-6 | 0.1 | 0.75 | 0.1 | insecticide | approved |
| Azinphos-methyl | 86-50-0 | 0.005 | 0.01 |  | insecticide | not approved |
| Bendiocarb | 22781-23-3 | 0.004 |  |  | insecticide | not approved |
| Benfuracarb | 82560-54-1 | 0.01 | 0.02 | 0.01 | insecticide | not approved |
| Bifenthrin | 82657-04-3 | 0.015 | 0.03 | 0.0075 | insecticide | approved |
| Bioresmethrin | 28434-01-7 | 0.03 |  |  | insecticide | not approved |
| Buprofezin | 953030-84-7 | 0.01 | 0.5 | 0.04 | insecticide | approved |
| Cadusafos | 95465-99-9 | 0.0004 | 0.003 | 0.0007 | insecticide | not approved |
| Carbaryl | 63-25-2 | 0.0075 | 0.01 | 0.01 | insecticide | not approved |
| Carbofuran | 1563-66-2 | 0.00015 | 0.00015 | 0.0003 | insecticide | not approved |
| Carbosulfan | 55285-14-8 | 0.005 | 0.005 | 0.005 | insecticide | not approved |
| Chlorfenapyr | 122453-73-0 | 0.015 | 0.015 |  | insecticide | not approved |
| Chlorfenvinphos | 470-90-6 | 0.0005 |  |  | insecticide | not approved |
| Chlorpyrifos | 2921-88-2 | 0.001 | 0.005 | 0.001 | insecticide | approved |
| Chlorpyrifos-methyl | 5598-13-0 | 0.01 | 0.1 | 0.01 | insecticide | approved |
| Chromafenozide | 143807-66-3 | 0.27 |  | 0.26 | insecticide | approved |
| Clothianidin | 210880-92-5 | 0.097 | 0.1 | 0.1 | insecticide | approved |
| Coumaphos | 56-72-4 |  |  |  | insecticide | not approved |
| Cyantraniliprol | 736994-63-1 | 0.01 |  | 0.007 | insecticide | approved |
| Cyfluthrin | 68359-37-5 | 0.003 | 0.02 | 0.02 | insecticide | not approved |
| Cyhalothrin, lambda- | 91465-08-6 | 0.0025 | 0.005 | 0.00063 | insecticide | approved |
| Cypermethrin | 52315-07-8 | 0.05 | 0.2 | 0.06 | insecticide | approved |
| Cypermethrin, zeta- | 52315-07-8 | 0.04 | 0.125 | 0.02 | insecticide | approved |
| Cyromazin | 66215-27-8 | 0.06 | 0.1 | 0.06 | insecticide | approved |
| Deltamethrin | 52918-63-5 | 0.01 | 0.01 | 0.0075 | insecticide | approved |
| Demeton-S-methyl | 919-86-8 |  |  |  | insecticide | not approved |
| Demeton-S-methyl-sulfon | 17040-19-6 |  |  |  | insecticide | not approved |
| Diazinon | 333-41-5 | 0.0002 | 0.025 | 0.0002 | insecticide | not approved |
| Dichlorvos | 62-73-7 | 0.00008 | 0.002 | 0.0005 | insecticide | not approved |
| Diflubenzuron | 35367-38-5 | 0.1 |  | 0.033 | insecticide | approved |
| Dimethoat | 60-51-5 | 0.001 | 0.01 | 0.001 | insecticide | approved |
| Dinotefuran | 165252-70-0 |  |  |  | insecticide | not approved |
| Emamectin | 155569-91-8 | 0.0005 | 0.01 | 0.0003 | insecticide | approved |
| Endosulfan | 115-29-7 | 0.006 | 0.02 |  | insecticide | not approved |
| EPN | 41198-08-7 | 0.03 | 1 |  | insecticide | not approved |
| Esfenvalerat | 66230-04-4 | 0.0175 | 0.0175 | 0.011 | insecticide | approved |
| Ethion | 563-12-2 | 0.002 |  |  | insecticide | not approved |
| Ethiprole | 181587-01-9 |  |  |  | insecticide | not approved |
| Ethoprophos | 13194-48-4 | 0.0004 | 0.01 | 0.001 | insecticide | approved |
| Etofenprox | 80844-07-1 | 0.03 | 1 | 0.06 | insecticide | approved |
| Fenitrothion | 122-14-5 | 0.005 | 0.013 |  | insecticide | not approved |
| Fenoxycarb | 72490-01-8 | 0.053 | 2 | 0.1 | insecticide | approved |
| Fenpropathrin | 39515-41-8 | 0.03 | 0.03 |  | insecticide | not approved |
| Fenthion | 55-38-9 |  |  |  | insecticide | not approved |
| Fenvalerat | 51630-58-1 | 0.0125 |  |  | insecticide | not approved |
| Fipronil | 120068-37-3 | 0.0002 | 0.009 | 0.0035 | insecticide | approved |
| Flonicamid | 158062-67-0 | 0.025 | 0.025 | 0.025 | insecticide | approved |
| Flubendiamid | 272451-65-7 | 0.017 | 0.1 | 0.006 | insecticide | approved |
| Flucythrinat | 70124-77-5 | 0.02 |  |  | insecticide | not approved |
| Flufenoxuron | 101463-69-8 | 0.01 |  | 0.01 | insecticide | not approved |
| Flupyradifurone | 951659-40-8 | 0.064 | 0.15 | 0.064 | insecticide | approved |
| HCH, gamma- (Lindan) | 58-89-9 |  |  |  | insecticide | not approved |
| Hydramethylnon | 67485-29-4 |  |  |  | insecticide | not approved |
| Imidacloprid | 138261-41-3 | 0.06 | 0.08 | 0.08 | insecticide | approved |
| Indoxacarb | 173584-44-6 | 0.006 | 0.125 | 0.004 | insecticide | approved |
| Isofenphos | 25311-71-1 | 0.001 |  |  | insecticide | not approved |
| Lufenuron | 103055-07-8 | 0.015 |  | 0.01 | insecticide | approved |
| Malathion | 121-75-5 | 0.03 | 0.3 |  | insecticide | approved |
| Metaflumizon | 139968-49-3 | 0.01 | 0.13 | 0.01 | insecticide | approved |
| Methacrifos | 62610-77-9 | 0.006 |  |  | insecticide | not approved |
| Methamidophos | 10265-92-6 | 0.001 | 0.003 |  | insecticide | not approved |
| Methidathion | 950-37-8 | 0.001 | 0.01 |  | insecticide | not approved |
| Methiocarb | 2032-65-7 | 0.013 | 0.013 | 0.013 | insecticide | approved |
| Methomyl | 16752-77-5 | 0.0025 | 0.0025 | 0.0025 | insecticide | approved |
| Methopren | 40596-69-8 |  |  |  | insecticide | not approved |
| Methoxyfenozid | 161050-58-4 | 0.1 | 0.2 | 0.1 | insecticide | approved |
| Methylbromid | 74-83-9 | 0.001 | 0.003 |  | insecticide | not approved |
| Metofluthrin | 240494-70-6 |  |  |  | insecticide | no information |
| Mevinphos | 7786-34-7 |  |  |  | insecticide | not approved |
| Milbemectin | 51596-10-2 | 0.03 | 0.03 | 0.014 | insecticide | approved |
| Monocrotophos | 6923-22-4 | 0.0006 | 0.002 |  | insecticide | not approved |
| Novaluron | 116714-46-6 | 0.01 |  |  | insecticide | not approved |
| Omethoat | 1113-02-6 | 0.0003 | 0.002 | 0.0003 | insecticide | not approved |
| Oxamyl | 23135-22-0 | 0.001 | 0.001 | 0.001 | insecticide | approved |
| Oxydemeton-methyl | 301-12-2 | 0.0003 | 0.0015 | 0.001 | insecticide | not approved |
| Parathion | 56-38-2 | 0.0006 | 0.005 |  | insecticide | not approved |
| Parathion-methyl | 298-00-0 |  |  |  | insecticide | not approved |
| Permethrin | 52645-53-1 |  |  |  | insecticide | not approved |
| Phenothrin | 26002-80-2 | 0.07 |  |  | insecticide | not approved |
| Phorat | 298-02-2 | 0.0007 | 0.003 |  | insecticide | not approved |
| Phosalon | 2310-17-0 | 0.01 | 0.1 |  | insecticide | not approved |
| Phosmet | 732-11-6 | 0.01 | 0.045 | 0.02 | insecticide | approved |
| Phosphamidon | 13171-21-6 | 0.0005 |  |  | insecticide | not approved |
| Piperonylbutoxid | 51-03-6 |  |  |  | insecticide | no information |
| Pirimicarb | 23103-98-2 | 0.035 | 0.1 | 0.035 | insecticide | approved |
| Pirimiphos-methyl | 29232-93-7 | 0.004 | 0.15 | 0.02 | insecticide | approved |
| Prallethrin | 23031-36-9 |  |  |  | insecticide | no information |
| Propoxur | 114-26-1 | 0.02 |  |  | insecticide | not approved |
| Pymetrozin | 123312-89-0 | 0.03 | 0.1 | 0.03 | insecticide | approved |
| Pyrethrine | 8003-34-7 | 0.04 | 0.2 |  | insecticide | approved |
| Pyridaben | 96489-71-3 | 0.01 | 0.05 | 0.005 | insecticide | approved |
| Pyridalyl | 179101-81-6 | 0.03 |  | 0.02 | insecticide | approved |
| Pyriproxyfen | 95737-68-1 | 0.1 |  | 0.04 | insecticide | approved |
| Spinetoram | 187166-40-1 | 0.025 | 0.1 | 0.0065 | insecticide | approved |
| Spinosad | 168316-95-8 | 0.024 |  | 0.012 | insecticide | approved |
| Spiromesifen | 283594-90-1 | 0.03 | 2 | 0.015 | insecticide | approved |
| Spirotetramat | 203313-25-1 | 0.05 | 1 | 0.05 | insecticide | approved |
| Sulfoxaflor | 946578-00-3 | 0.04 | 0.25 | 0.06 | insecticide | approved |
| tau-Fluvalinat | 102851-06-9 | 0.005 | 0.05 | 0.0044 | insecticide | approved |
| Tebufenozid | 112410-23-8 | 0.02 |  | 0.008 | insecticide | approved |
| Teflubenzuron | 83121-18-0 | 0.01 |  | 0.016 | insecticide | approved |
| Tefluthrin | 79538-32-2 | 0.005 | 0.005 | 0.0015 | insecticide | approved |
| Terbufos | 13071-79-9 |  |  |  | insecticide | not approved |
| Thiacloprid | 111988-49-9 | 0.01 | 0.03 | 0.02 | insecticide | approved |
| Thiamethoxam | 153719-23-4 | 0.026 | 0.5 | 0.08 | insecticide | approved |
| Thiodicarb | 59669-26-0 | 0.01 | 0.01 |  | insecticide | not approved |
| Tolfenpyrad | 129558-76-5 |  |  |  | insecticide | not approved |
| Transfluthrin | 118712-89-3 |  |  |  | insecticide | no information |
| Triazamate | 112143-82-5 |  |  |  | insecticide | not approved |
| Triazophos | 24017-47-8 | 0.001 | 0.001 |  | insecticide | not approved |
| Trichlorfon | 52-68-6 | 0.045 | 0.1 | 0.09 | insecticide | not approved |
| Triflumuron | 64628-44-0 | 0.014 |  | 0.036 | insecticide | approved |
| Vamidothion | 2275-23-2 | 0.008 |  |  | insecticide | not approved |
| 1,4-Dimethylnaphtalene | 571-58-4 | 0.1 |  | 0.32 | plant growth regulator | approved |
| 2-(1-naphthyl)acetamide | 86-86-2 | 0.1 | 0.1 | 0.07 | plant growth regulator | approved |
| 2-(1-naphthyl)acetic acid | 86-87-3 | 0.1 | 0.1 | 0.07 | plant growth regulator | approved |
| 2-Naphtyloxyacetic acid | 120-23-0 | 0.01 | 0.6 | 0.03 | plant growth regulator | not approved |
| 6-Benzyladenin | 1214-39-7 | 0.01 |  | 0.03 | plant growth regulator | approved |
| Chlormequat | 999-81-5 | 0.04 | 0.09 | 0.04 | plant growth regulator | approved |
| Clofencet | 129025-54-3 |  |  |  | plant growth regulator | not approved |
| Cyclanilid | 113136-77-9 | 0.0075 | 0.015 | 0.0045 | plant growth regulator | not approved |
| Daminozid | 1596-84-5 | 0.45 |  | 0.16 | plant growth regulator | approved |
| Dichlorbenzoate-methylr | 2905-69-3 |  |  |  | plant growth regulator | approved |
| Dimethipin | 55290-64-7 |  |  |  | plant growth regulator | not approved |
| Ethephon | 16672-87-0 | 0.03 | 0.05 | 0.03 | plant growth regulator | approved |
| Flumetralin | 62924-70-3 | 0.015 | 0.1 | 0.03 | plant growth regulator | approved |
| Flurprimidol | 56425-91-3 | 0.003 | 0.09 | 0.003 | plant growth regulator | not approved |
| Forchlorfenuron | 68157-60-8 | 0.05 | 1 | 0.25 | plant growth regulator | approved |
| Indolylbutyric acid | 133-32-4 |  |  |  | plant growth regulator | approved |
| Maleinsäurehydrazid | 10071-13-3 | 0.25 |  | 0.25 | plant growth regulator | approved |
| Mepiquat chlorid | 24307-26-4 | 0.2 | 0.3 | 0.3 | plant growth regulator | approved |
| Paclobutrazol | 76738-62-0 | 0.022 | 0.1 | 0.1 | plant growth regulator | approved |
| Prohexadione | 127277-53-6 | 0.2 |  | 0.35 | plant growth regulator | approved |
| Sintofen | 130561-48-7 | 0.091 |  | 0.165 | plant growth regulator | approved |
| Trinexapac-ethyl | 95266-40-3 | 0.32 |  | 0.34 | plant growth regulator | approved |
| Acequinocyl | 57960-19-7 | 0.023 | 0.08 | 0.014 | acaricide | approved |
| Acrinathrin | 101007-06-1 | 0.01 | 0.01 | 0.007 | acaricide | approved |
| Azocyclotin | 41083-11-8 | 0.003 | 0.02 |  | acaricide | not approved |
| Bifenazat | 149877-41-8 | 0.01 |  | 0.0028 | acaricide | approved |
| Bromopropylat | 18181-80-1 | 0.03 |  |  | acaricide | not approved |
| Clofentezin | 74115-24-5 | 0.02 |  | 0.01 | acaricide | approved |
| Cyflumetofen | 400882-07-7 | 0.17 |  | 0.11 | acaricide | approved |
| Cyhexatin | 13121-70-5 | 0.003 | 0.02 |  | acaricide | not approved |
| Dicofol | 115-32-2 | 0.002 |  |  | acaricide | not approved |
| Etoxazol | 153233-91-1 | 0.04 |  | 0.03 | acaricide | approved |
| Fenazaquin | 120928-09-8 | 0.005 | 0.1 | 0.01 | acaricide | approved |
| Fenbutatin Oxid | 13356-08-6 | 0.05 | 0.1 |  | acaricide | not approved |
| Fenpyroximate | 134098-61-6 | 0.01 | 0.02 | 0.005 | acaricide | approved |
| Flumethrin | 69770-45-2 |  |  |  | acaricide | no information |
| Formetanat | 23422-53-9 | 0.004 | 0.005 | 0.004 | acaricide | approved |
| Hexythiazox | 78587-05-0 | 0.03 |  | 0.009 | acaricide | approved |
| Propargit | 2312-35-8 |  |  |  | acaricide | not approved |
| Spirodiclofen | 148477-71-8 | 0.015 |  | 0.009 | acaricide | approved |
| Tebufenpyrad | 119168-77-3 | 0.01 | 0.02 | 0.01 | acaricide | approved |
| Fenamiphos | 22224-92-6 | 0.0008 | 0.0025 | 0.0008 | nematicide | approved |
| Fluensulfon | 318290-98-1 |  |  |  | nematicide | no information |
| Fosthiazat | 98886-44-3 | 0.004 | 0.005 | 0.005 | nematicide | approved |
| DDAC | 7173-51-5 |  |  |  | molluscicide | not approved |
| Metaldehyd | 108-62-3 | 0.02 | 0.3 | 0.1 | molluscicide | approved |
| Bronopol | 52-51-7 | 0.02 |  |  | bactericide | not approved |
| Cyprosulfamid | 221667-31-8 |  |  |  | herbicide safener | not approved |
| Kasugamycin | 6980-18-3 |  |  |  | bactericide | not approved |
| Picaridin | 119515-38-7 |  |  |  | insect repellent | no information |
| Bromadiolone | 28772-56-7 |  |  | 0.0000012 | rodenticide | approved |
| Temephos | 3383-96-8 |  |  |  | insect attractant | not approved |
| Propylenethiourea | 51-52-5 |  |  |  | carbamate metabolite | no information |
| Quinoclamine | 2797-51-5 | 0.002 | 0.05 | 0.03 | algicide | approved |
